# Opportunities and Challenges of a Self-Management App to Support People With Spinal Cord Injury in the Prevention of Pressure Injuries: Qualitative Study

**DOI:** 10.2196/22452

**Published:** 2020-12-09

**Authors:** Julia Amann, Maddalena Fiordelli, Anke Scheel-Sailer, Mirjam Brach, Sara Rubinelli

**Affiliations:** 1 Swiss Paraplegic Research Nottwil Switzerland; 2 Health Ethics and Policy Lab Department of Health Sciences and Technology ETH Zurich Zurich Switzerland; 3 Institute of Communication and Health Faculty of Communication Sciences Università della Svizzera italiana Lugano Switzerland; 4 Swiss Paraplegic Center Nottwil Switzerland; 5 Department of Health Sciences and Medicine University of Lucerne Lucerne Switzerland

**Keywords:** mHealth, eHealth, self-management, spinal cord injury, pressure injury, prevention, technology acceptance

## Abstract

**Background:**

Mobile health applications can offer tailored self-management support to individuals living with chronic health conditions. However, there are several challenges to the adoption of these technologies in practice. Co-design is a promising approach to overcoming some of these challenges by enabling the development of solutions that meet the actual needs and preferences of the relevant stakeholder groups.

**Objective:**

Taking spinal cord injury as a case in point, the overall objectives of this study were to identify the perceived benefits of a co-designed self-management app that could promote its uptake and to explore the factors that may impede adoption.

**Methods:**

We adopted a qualitative research approach guided by the Technology Acceptance Model. Data were collected through semistructured interviews with individuals with spinal cord injury (n=15) and two focus groups with health care professionals specialized in spinal cord injury (n=7, n=5). Prior to the interviews and focus groups, study participants were given time to explore the app prototype. All interviews were transcribed verbatim and analyzed using inductive thematic analysis.

**Results:**

Findings of our analysis indicate that study participants perceived the app prototype as potentially useful for supporting individuals with spinal cord injury in preventing pressure injuries. In particular, we identified three concrete use cases highlighting the benefits of the app for different audiences: (1) a companion for newly injured individuals, (2) an emergency kit and motivational support, and 3) a guide for informal caregivers and family members. We also uncovered several challenges that might impede the adoption of the self-management app in practice, including (1) challenges in motivating individuals to use the app, (2) concerns about the misuse and abuse of the app, and (3) organizational and maintenance challenges.

**Conclusions:**

This study adds to a growing body of research that investigates individuals’ adoption and nonadoption behavior regarding mobile health solutions. Building on earlier work, we make recommendations on how to address the barriers to the adoption of mobile health solutions identified by this study. In particular, there is a need to foster trust in mobile health among prospective users, including both patients and health care professionals. Moreover, increasing personal relevance of mobile health solutions through personalization may be a promising approach to promote uptake. Last but not least, organizational support also plays an instrumental role in mobile health adoption. We conclude that even though co-design is promoted as a promising approach to develop self-management tools, co-design does not guarantee adoption. More research is needed to identify the most promising strategies to promote the adoption of evidence-based mobile health solutions in practice.

## Introduction

Mobile health applications promise to provide tailored self-management support to individuals living with chronic health conditions. However, there are several challenges to the adoption of these technologies in practice. First, while there is an abundance of health apps freely available, the majority are not evidence-based [1,2]. This is especially true for apps aimed at individuals with disabilities [3-5]. There are also no clear guidelines or quality standards when it comes to mobile health (mHealth) applications. This may, in turn, discourage health care professionals from recommending them to patients [6]. Second, research has shown that individuals’ use of health applications often declines over time when the initial phase of curiosity has worn off [7,8]. A potential explanation for this may be that there is a misfit of the technology leading to a lack of integration into the person’s life. Third, technologies are evolving at an ever-increasing pace, yet, in health care, moving from development to adoption is a long and complex process [9,10]. This means that once a pilot evaluation is concluded, the application’s design and functionalities may already be outdated and may thus fail to reproduce the outcomes of a pilot evaluation in a real-life setting.

Involving relevant stakeholder groups in the early stages of the app development process constitutes a promising approach to address these challenges [11-14]. First, co-design can ensure an evidence-guided approach to translating different stakeholders’ needs and preferences into digital solutions in a meaningful way [12,15-19]. Moreover, if decisions regarding content, functionality, and design are guided by real-life experiences of prospective users, this helps to ensure the appropriateness and relevance of self-management applications. Involving prospective users also fosters trust and the credibility of the technology, thus increasing the likelihood of system acceptance and, as a result, accelerating adoption into practice [20,21].

This study forms part of a larger co-design research project carried out in Switzerland. The project aimed to develop an evidence-based self-management app to support community-dwelling individuals with spinal cord injury (SCI) in the prevention of pressure injuries. SCI is a complex chronic health condition that makes individuals prone to incurring secondary health conditions. Pressure injuries are among the most common and serious secondary health conditions in people with SCI [22-24]. In addition to a number of nonmodifiable risk factors (eg, natural skin aging) [25], self-management plays a key role in the prevention of pressure injuries [26,27]. The overall objectives of this study were to identify the perceived benefits of the self-management app that could promote its uptake and to explore the factors that may impede its adoption.

## Methods

### Study Design

**Figure 1:**
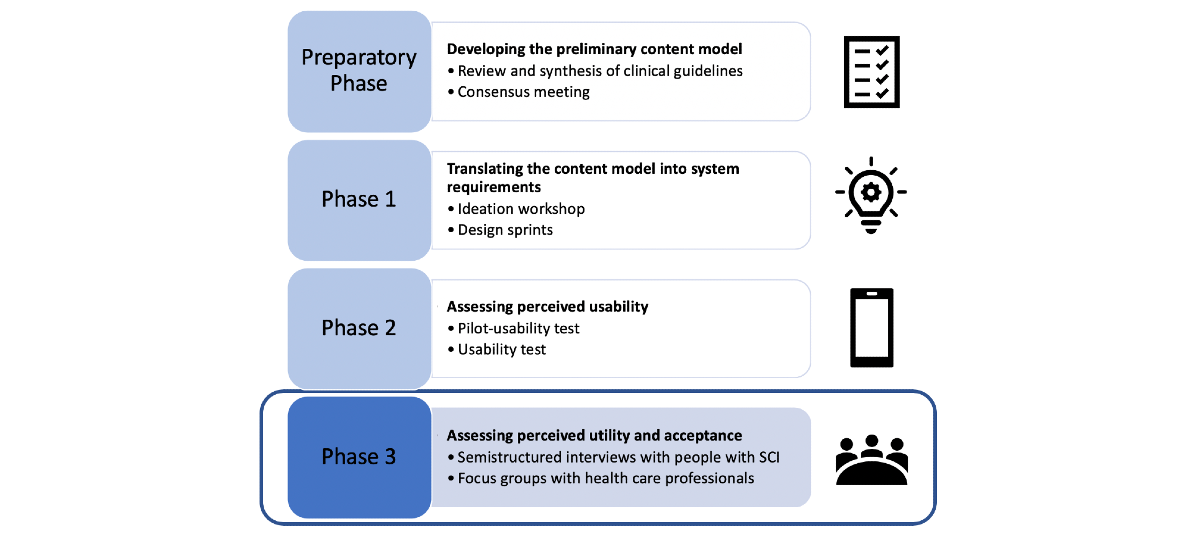
Overview of the study methodology. SCI: spinal cord injury.

This study forms part of a larger mixed-methods study that combined qualitative research methods (ie, semistructured interviews) into user-centered design approaches (ie, ideation workshop, design sprints, usability tests). Figure 1 illustrates the methodological approach of the overall project. Detailed descriptions of the app development process have been published elsewhere [5,28]. This paper focuses on assessing the perceived utility and acceptance of the app prototype that resulted from the previous phases of this project. The study was approved by the cantonal ethics commission (EKNZ 2017-01787).

### Data Collection and Analysis

Following a convenience sampling approach, we conducted 15 semistructured interviews with individuals with SCI and two focus groups with health care professionals specialized in SCI (n=7; n=5). Both individuals with SCI and health care professionals were given time to explore the app prototype (see Multimedia Appendix 1 for screenshots of the app prototype) prior to the interviews/focus groups. People with SCI first took part in a usability test [28] that allowed them to explore the app prototype in more depth. Healthcare professionals received a brief introduction to the main functionalities and elements of the app by the first author (JA). They could then freely explore the app prototype in pairs for 15 minutes before the focus group discussion. The interview and focus group guides were informed by the constructs of the Technology Acceptance Model [29]. In particular, we aimed to capture participants’ views regarding usability and perceived usefulness of the app prototype, as well as their attitudes and intentions toward using the app. In this paper, we focused our analysis on identifying concrete benefits of the app that could promote its uptake and exploring the factors that may impede its adoption into practice. Results of a preliminary usability test of the co-designed self-management app are reported elsewhere [28].

All interviews and both focus groups were conducted in German by the first author (JA), who is a native German speaker and communication scholar trained in qualitative research methods. All interviews and both focus groups were audio-recorded. Additionally, a research assistant was present during all interviews and focus groups to take notes. Interviews lasted 33 minutes on average, ranging between 17 and 69 minutes. Focus group 1 (FG1; n=7) lasted 100 minutes, and focus group 2 (FG2; n=5) lasted 97 minutes. In total, 687 minutes of audio material were transcribed verbatim for analysis.

Data were analyzed using inductive thematic analysis [30]. For this purpose, initial line-by-line coding was carried out by the first author (JA). In this initial coding phase, we aimed to identify instances where participants described situations where they either would personally use or could imagine someone else engaging with the self-management app. In the next step, we focused on uncovering the factors that may impede adoption by focusing on those instances where participants voiced concerns or challenges. Starting from the initial coding and in line with our research objectives, we identified preliminary themes and subthemes, which were continuously refined through discussion in regular meetings involving all coauthors.

### The App Prototype

The interactive web-based app prototype evaluated in this study is the result of a multistage co-design process [5,28]. It includes five main features: (1) a smart camera, (2) a pressure injury diary, (3) an expert consultation, (4) reminders, and (5) a knowledge repository.

As such, the app prototype comprises features that can be used independently by people with SCI (eg, documenting pressure injuries, setting reminders), as well as a communication component that allows for interaction with Parahelp, a home care service provider specialized in SCI care. (See Multimedia Appendix 1 for screenshots of the app prototype.)

### Participants

Within the scope of the overall project, we aimed to involve a balanced sample of participants in terms of age, gender, lesion level for people with SCI, and health care professionals’ expertise [28]. Prospective participants were identified through the local rehabilitation center (Swiss Paraplegic Center), patient association (Swiss Paraplegic Association), and home care provider specialized in SCI (Parahelp). Additionally, we launched a call for participants on the Paraplegie Community [31], an online community for people with SCI initiated and funded by the Swiss Paraplegic Foundation. All participants received detailed information on the study and signed a consent form. Study participants' characteristics for the interviews and focus groups are presented in Table 1 and Table 2, respectively.

**Table 1:** Interview participant characteristics.

Characteristic			People with SCI (n=15)
Age (years), mean (range)			40.8 (28-58)
**Gender, n**			
	Male	11	
	Female	4	
**Lesion level, n**			
	Paraplegic	7	
	Quadriplegic	8	

**Table 2:** Focus group participant characteristics.

Characteristic		Total (n=12)	Focus group 1 (n=7)	Focus group 2 (n=5)
Work experience (years), mean (range)		22.9 (10-36)	25.4 (12-36)	19.4 (10-30)
**Gender, n**				
	Male	1	1	0
	Female	11	6	5
**Professional role, n**				
	Wound expert	7	5	2
	Administrator	1	1	0
	Psychologist	1	0	1
	Occupational therapist	1	0	1
	Nutritionist	1	1	0
	Therapy instructor	1	0	1

## Results

### Overview

Findings of our analysis indicate that both individuals with SCI and health care professionals saw potential value in the self-management app prototype. Study participants acknowledged its usefulness for different life situations and target groups, including newly injured individuals and experienced wheelchair users, as well as informal caregivers. In addition, we also identified some of the key factors that could impede the app’s adoption into practice. In the Results section, we illustrate our findings using quotes from the interviews and focus groups, each followed by a participant identifier. With the exception of the theme “Organizational and maintenance challenges” (which was predominantly present in the focus groups with health care professionals), themes occurred fairly equally in both groups. Thus, when we refer to “study participants,” this encompasses both individuals with SCI and health care professionals.

### Benefits Promoting Uptake

#### A Companion for Newly Injured Individuals

Wheelchair users and health care professionals concurred that individuals who recently suffered a spinal cord injury, for example, those who are completing or have recently completed first rehabilitation, could particularly benefit from the app as a complementary measure to traditional patient education.

The app could be complementary [to traditional patient education], yes. So the nurses could introduce the app to patients, maybe those that are not so much into paper-based information sheets and prefer something more interactive.

B, FG2

One of the participants with SCI underlined how the app could support people who were recently injured in processing information at their own pace and in their own time. In recalling her own experiences from first rehabilitation, the participant described how the app could contribute to fostering patient autonomy and self-determination.

But then the problem is simply that you were forced to attend this information day [on pressure injuries]. And with an app like this, I have the feeling that you have a look at the information when you want to. And you take as much information as you can process at that particular moment. And the advantage is simply that I can just shut it down. And I can then, perhaps in the evening, have another look at it when I'm in bed. I can really adjust it to my pace. And then, I feel, you're more receptive. Because everybody has their own pace. And I think it's bad when a certain pace is forced on you. Even on important issues. But if it's forced on me and I block it out, then it's no good for me.

P15

Another important aspect that participants noted was that the app could help to lower individuals’ inhibition thresholds when in need of medical assistance or advice. In other words, they described the app as a low-key means of getting in touch with evidence-based knowledge and a health care professional at times or in situations where one might otherwise feel uncomfortable. Given that newly injured individuals in particular may not immediately recognize the seriousness of an early-stage pressure injury or may be embarrassed to seek help, participants noted that the app could support these individuals in early detection before the situation worsens.

Especially on Sunday or late in the evening, then you don't dare to call, then you can just quickly ask, I think that's a very good thing. For example, if you get a bladder infection with a fever and you don't know exactly how/what/where - if you don't have that much experience. Certainly also for the relatives or caregivers [this would be useful]...at the onset, I think that's great.

P2

Or you can also ask the question anonymously if you are embarrassed, if the pressure injury is in an embarrassing place, for example. [Then it's probably easier to ask such a question], I could imagine. Maybe if you're embarrassed about having to take a picture [of a pressure injury] on your butt.

P6

Both individuals with SCI and health care professionals also pointed out that the app could help newly injured individuals to build and adopt new habits to better self-manage. Building these new habits after partial loss of functioning is key to effective self-management. Wheelchair users attributed particular value to the reminder function as a potentially powerful habit-building assistance that can provide structure and introduce new routines into someone’s life.

I would target first rehab patients more. The ones who don't have the knowledge are the ones who really need to be told. So that it becomes routine.

P13

I should be able to work with that, but maybe someone who's just starting rehab. ...They'd have to use the app to get into the rhythm. That helps 100% in my opinion.

P1

So, to some extent, if you get the reminder, maybe it can help you that you get into a rhythm like, like I have developed one now over the years. And you say, “Okay, over lunchtime, in the evening I look at this and that,” and yes, maybe that can help you. That in the end you'll have the rhythm internalized. You just get the reminder. So just as a support. Because I think that's important. That you still have a rhythm of your own, but you can adjust this here [in the app].

P11

#### An Emergency Kit and Motivational Support

Individuals with SCI highlighted that the app could be particularly useful under extraordinary conditions (ie, outside of their daily routine). According to them, these were situations where even experienced wheelchair users might be hesitant or unable to use their usual means of contact (ie, telephone call). Frequently mentioned examples of such extraordinary circumstances were during holidays abroad, where one might not trust the foreign health care system, or when fallen ill. Wheelchair users also described nighttime and weekends as situations where the app could potentially come in handy.

You can directly get in touch with people, with specialists, I would appreciate that. For example, I once had a bruise when I lived in the south of France, then I actually did it exactly like that: I took pictures and thought about which doctors I knew and sent the photo.

P10

If it looks like this, you should try this and that for two days, and if it doesn't get better, you have to do something. Now if someone didn't have a doctor while they are on holiday, let's say, if you're somewhere where you don't have that much confidence in medical assistance, I think it [the app] is great.

P6

These views were shared by the health care professionals. They also noted that the app could support wheelchair users in recognizing skin problems at an early stage. According to them, the app could help to reduce the risk of individuals waiting too long to get in touch by providing them with easy access to health care providers’ expertise. In this context, health care professionals emphasized the benefits of being able to receive images (ie, using the smart camera feature), allowing them to get an idea of how serious the situation is.

A: Maybe this app would lower the inhibition threshold to ask questions. So if someone has the feeling “Ah, I have a scratch or something” and before going running to the doctor or saying “[It's not that bad,] I'm not going for just that,” you could ask experts, send a photo. I think maybe this could help you to identify [a skin problem] early or act or react faster.

B: That's for at home, when uncertainties arise, when you don't know exactly what you're supposed to do. So you can just quickly send a photo to Parahelp.

D: Yes or maybe make an appointment and talk...

B: Right, instead of having to drive 30 km [to see a health care professional in person].

FG2

Both wheelchair users and health care professionals described refreshing one’s memory and extending knowledge on pressure injury prevention as key benefits that could promote self-management, particularly among more experienced wheelchair users. In addition, being able to learn from one’s own experience by reflecting on past experiences or behaviors (ie, through self-documentation in the pressure injury diary) was mentioned as a potential motivational boost to become more self-aware and attentive.

You can experiment a little, browse around a little. Maybe refresh some forgotten knowledge. Watch a short video. How do others do it? The curiosity “What do the others do?” is always there, so it's actually a win-win situation.

FG1, D

I say now, long-term reminders I like because, for example, I didn't know that there was such thing as a cushion-check. I would do it if I no longer felt comfortable or was no longer sitting comfortably, then I would do a cushion-check. This can sometimes take two to three days or maybe even a weekend. I do a lot intuitively, so it's good to introduce certain things like the cushion-check, I think that's a good thing.

P1

Here I have the opportunity to see this every day when a photo comes in. To see what I should change, have I worn a different shoe, or are was it a hot day and the foot is swollen. That would be more obvious here, in any case. I could go through this process more thoroughly [in the app], in the sense of self-reflection. Through reflection we learn, we can also develop and grow. That has certain advantages, I think.

P9

#### A Guide for Informal Caregivers and Family Members

According to the study participants, informal caregivers could also benefit from using the app as a tool to learn more about spinal cord injury and the risk of pressure injuries. They suggested that the app could allow caregivers to better understand the manifold challenges that individuals with SCI face and propose ways to better support them in their daily life. In this context, both wheelchair users and health care professionals mentioned practical aspects that could help to increase knowledge and skills among caregivers and, as a result, improve support for people with SCI. In their remarks, participants touched upon the importance of shared responsibility of care and the related need to provide caregivers with evidence-based information and practical support.

Yes, or a caregiver. I'd say a 60+ elderly lady, who looks after her husband. So if a person is really sleeping all day, that you remind the caregiver or the person themselves: “Okay, I have to change sides or lie on my stomach.”

P14

And also, and certainly, the family members. The next of kin. That they know what this is all about. Because it's not enough for the person in the wheelchair to know, instead the family should also know where the spot is and know if it's somehow normal-red, which means it can be pressed away.

P15

Above all, however, we notice that the relatives are most interested in what could change or has changed in terms of their [wheelchair user's] care and status, at least that's the case for first-rehab patients. Those [informational materials] are mostly used by the relatives, also at home, when suddenly questions arise that have not yet been a topic in everyday life.

D, FG2

When comparing how information was currently provided to family members (ie, a bulky informational brochure) to the possibilities that the app would offer, health care professionals agreed that it would make information retrieval easier. They argued that an app could easily be used on the go, at any time, even just out of boredom. According to them, this may not only help family members to save time when looking for information but may also incentivize them to do so. Most importantly, the app would provide them with curated, evidence-based information.

B: Yes, that's just the way it is, they hear, the relatives also hear everything, but it's perhaps a lot of information on such a day and yes...That's the issue with the patient manual, it's just an A4 folder, a fairly bulky folder that takes up space. You always have to open it first...And just like that, here [with the app] open it and bang, bang, bang, and then you are where you are...That's the difference maybe, which makes it more motivating to deal with it.

G: You're probably more likely to have a look at it.

B: Yes, when you're bored and then just browse.

G: You click around more.

F: It always saves time when I am looking for information.

FG2

One study participant with SCI also referred to the potential of the app to address emotional aspects that are of great importance for the psychological well-being of people with SCI and the people around them alike. In his view, the app could help to foster understanding and empathy for individuals with SCI.

Yes, for all those in wheelchairs and this could also be interesting for the family, provide a clear overview, or for people who are not directly but indirectly affected through someone they know. That they get help and tips on how to interact with my son or friend, that's what I find super cool. ...because a short example, I thought I had a huge family but since my accident three years ago they have all disappeared. So really disappeared or they are distancing themselves, etc.

P5

### Challenges Impeding Adoption

#### Motivating Individuals to Use the App

Established attitudes and behavioral patterns of people with SCI toward pressure injuries constitute potential obstacles. A key challenge we identified relates to onboarding or, in other words, motivating both experienced and newly injured individuals to use the app. Healthcare professionals voiced concerns that wheelchair users may, in fact, only start using the app once they experience skin problems. This raises questions as to how to best engage newly injured individuals in early preventive measures.

There are many who come and say: Now I've been in a wheelchair for 20 years, I've had no problems with my skin and now all of a sudden I have a decu [pressure injury]. Well, that won't be someone who has regularly looked into such an app before. You probably only do that once you experience problems.

C, FG2

I see it very much as an acute approach, “If something happens to me, I have a red spot, uh. I know here [in the app] I might find an answer.“

F, FG2

In line with this, some of the wheelchair users noted that they didn’t consider themselves a primary target audience. In this context, some noted that frequent reminders in the form of pop-up messages could easily become annoying. Drawing on their own experiences, wheelchair users explained that they had internalized important preventive behaviors and knowledge. While they could see the potential benefits of the app for others, both newly injured and experienced, they themselves indicated that they would not need the app.

The reminders can also become too much, but in a way, I think it's actually good, because sometimes it takes a bit [of a push], doesn't it? Many, especially wheelchair users, who may have other things to do...It helps them if they are reminded a little bit of what they have to do, of things that might not be a lot of fun right now. But maybe it will give them the extra drive when you see it [the reminder]. And then you do it and then you're done.

P3

I don't really need reminders as a support, I actually do everything quite independently. Sometimes you notice that it wasn't enough pressure-release and that you have to do something else again - so, no, I don't need something like that [an app to remind me].

P2

Similarly, the secure contact function was viewed as beneficial, yet many wheelchair users noted that under ordinary conditions, they would rather see a health professional in person or call them in case of an emergency. A key consideration in case of an emergency was to choose the quickest and simplest way to get professional assistance, and according to some, this may not necessarily be through the app’s contact function.

Many people are afraid [to call]. But with me, if I want to know something, I usually call quickly. And I know I can find it somewhere on the website myself, but I don't have time. Just tell me quickly and done.

P4

If I had a pressure injury, I'd take a picture of it. I had one once and I was told to take a picture of it when I called. My friend took a picture of it quickly. The question is, how fast will it be handled when I put it into an app or when I call, what is the better or faster way, that's what I'm wondering.

P6

In relation to behavioral and attitudinal barriers, health care professionals raised another important issue they encountered in their daily work: ”regular clients“ (ie, patients who regularly have problems and often have to be hospitalized because of these problems). According to them, this group of patients would use pressure injuries as a means of attracting attention, sometimes even consciously manipulating wounds to receive care. They considered it unlikely that these individuals could be motivated to use the app, let alone benefit from the app to prevent pressure injuries.

B: I know wound patients who tamper with their wounds so that someone will come and take care of them.

A: Exactly, but unfortunately we can't reach those people [with the app].

B: But these are not necessarily the people who want to improve their situation. But these are the people who, let's say, when they experience healing, then they also experience a loss of care and this loss of care they just can't handle...So these are not the people we reach with something like that [the app].

FG2

#### Concerns About Misuse and Abuse of the App

Study participants expressed concerns that the app could potentially be misused and that both patients and health care professionals could fall victim to such misuse. On the one hand, they worried about privacy and data protection issues that prospective app users would be facing. In particular, participants raised questions regarding the storage, sharing, backup, and deletion of personal data.

On the one hand, that would be an advantage for me and certainly also an advantage if you could read it again. On the other hand, I simply have to ask myself, is the data even stored safely? Does it really stay where it is or is it passed on to third parties?

P12

Privacy is very important to me that people keep information to themselves and don't pass it on to anyone. ...Inside Parahelp I think it would be ok, but if it suddenly ends up with the plastic surgeons, I would really have a problem with that. It must really be a trusting relationship.

P13

If I delete the app again, I have seen that there is a registration, so I assume that it [data] remains stored there. Do I want to have this data available or do I want it effectively deleted so that it is no longer on the server or delete the entire registration? That would be interesting to know. Yes, the risk is, that something is done with the data. ...

P14

However, these concerns were not shared by all participants. For instance, one study participant with SCI noted that the stand-alone use of a smartphone with mobile data enabled would already compromise personal privacy. He thus did not consider the collection of data through the self-management app problematic but rather a necessary requirement to provide adequate support.

Either you live totally cut off or you can forget it because as soon as you have even one Facebook account, or just one Instagram account, or a smartphone with data volume, your privacy is limited. That's why it [the app] is not dangerous at all, on the contrary...The data helps you, that's why it needs certain permissions, like camera access, like audio access and position navigation, like GPS data coordinate access, that's what it needs.

P5

In addition to privacy concerns impacting the user, some study participants were concerned about the potential misuse of the app by patients and the negative impact this could have on health care professionals. In particular, they worried about excessive messaging from people with SCI without actual skin problems. According to the study participants, the fact that it would easier to initiate contact may lower individuals’ inhibition thresholds, thus increasing the risk that some people might contact health care professionals for irrelevant information or just to talk to someone.

Maybe lonely people who put a lot of stress on the experts, that would be a disadvantage for the experts. Yes, there are many wheelchair users who are lonely. You can see that around here [specialized center], there are many who want to live here and try to get something [a place to live here]. Here it's a place where you don't feel lonely anymore. I can imagine that if you are bored, some people will just have a look [at who is online]. “There's Julia or Freddy. I'll ask.”

P10

The fact is, there are many who feel alone and they just want to chat and start [a conversation] for every little thing or for nothing. But the aim is certainly not to overwhelm the professionals.

P5

The risk is just that you have people out there who are just lonely and would use the app in search of interaction, you know?

F, FG1

#### Organizational and Maintenance Challenges

When discussing what was needed to bring the app into practice, health care professionals also referred to organizational problems, including the difficulty of integrating the app with currently used systems. They feared that the adoption of the app might increase their workload (eg, having to reenter existing information). Healthcare professionals were also unsure of how to securely identify their clientele and worried about managing resources. Here, it is important to note that unlike other home care providers, Parahelp has a specific care mandate, meaning that only individuals meeting predetermined criteria are entitled to receive assistance from Parahelp (ie, persons with spinal cord injury). Determining whether these conditions are met was highlighted as a core challenge by health care professionals.

You have to be able to make a query to determine: does this person belong to our target audience or not. Because if you can't have a professional make the selection, then we'll suddenly have everyone in the chat. And then it [workload] explodes.

F, FG1

With regard to the challenges of integrating the app into practice, some health care professionals also pointed to weaknesses in the app's content. For example, the lack of information on psychological aspects was criticized. The maintenance and further development of the app were also heavily discussed. In particular, health care professionals were concerned about how information would be kept up to date and who would be responsible for ensuring information accuracy. They agreed that the app content should be regularly checked to ensure that it contains the latest evidence-based information. However, it was unclear who would be responsible for this task.

Exactly, so I think the possibility for developments and new innovations - because this is also evolving- this would need to be included [in the app]. It just has to be taken up somehow.

B, FG2

A: But then who feeds the whole thing [app] with topics and content? So if I have such a question now as someone affected, that I go to Romania or something like that? Or I have the question...

B: It won't be that specific...

A: Somebody has to feed [content into] that [the app].

B: Right, exactly. Of course, it can't be that specific, you can't provide resources for every country, it's not possible, but if for example, the person goes to a hot country, or to a cold country.

FG2

## Discussion

### Principal Findings

This study identified the perceived benefits of a co-designed self-management app for the prevention of pressure injuries that could promote its uptake and shed light on the factors that may impede its adoption in practice. It is one of the first studies on the perceived opportunities and challenges of a mobile health application to support individuals with spinal cord injury in the prevention of pressure injuries. By capturing both the perspectives of people with spinal cord injury and health care professionals, it complements earlier work, which has predominantly focused on assessing patient views [32] and compliance with app-based interventions [33].

Study participants perceived the app as potentially useful for supporting individuals with SCI in preventing pressure injuries. In particular, we identified three concrete use cases highlighting the benefits of the app: (1) a companion for newly injured individuals, (2) an emergency kit and motivational support, and (3) a guide for informal caregivers and family members. In addition, we also uncovered several challenges that might impede the adoption of the self-management app in practice, including (1) challenges in motivating individuals to use the app, (2) concerns about the misuse and abuse of the app, and (3) organizational and maintenance challenges.

### Fostering the Adoption of Self-management Apps

**Figure 2:**
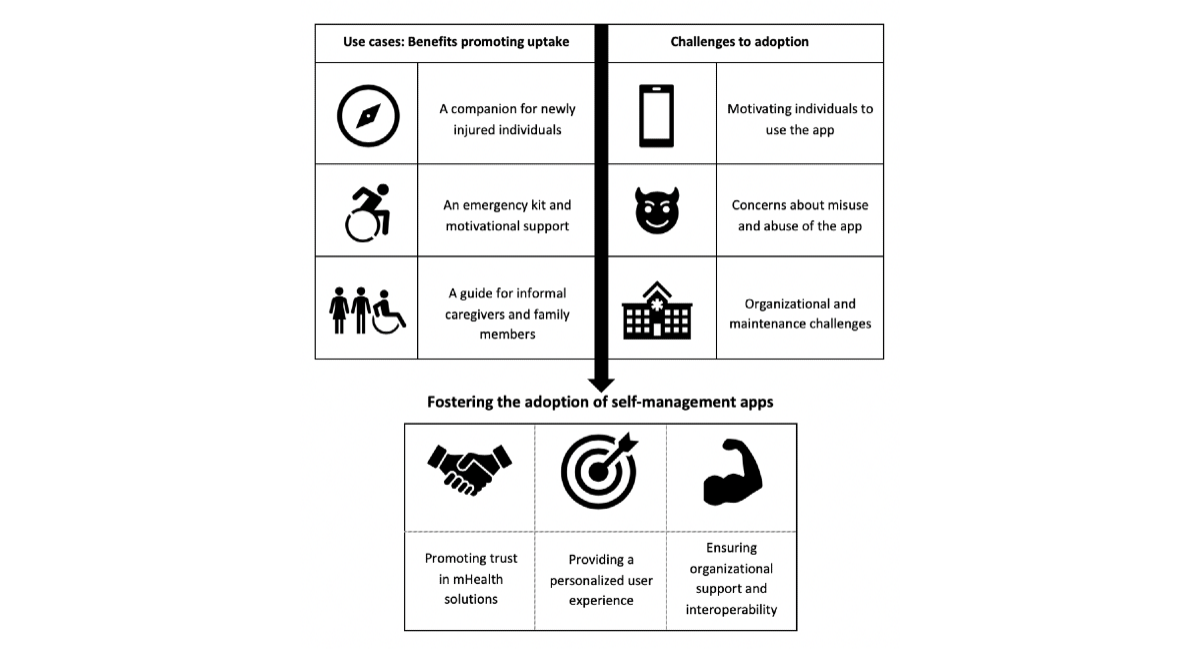
Fostering the adoption of self-management apps.

It seems intuitive that a self-management app can be experienced as empowering and motivating by some while being deemed useless or even annoying by others. Similarly, some health care professionals may welcome an app as a promising alternative, while others may worry about increased workload. What can be done to capitalize on the app’s perceived benefits and to reduce barriers to uptake? Several studies have addressed this question more generally by developing frameworks aimed at improving the uptake and impact of eHealth technologies [14,34]. Drawing on our findings and Greenhalgh et al’s nonadoption, abandonment, scale-up, spread, and sustainability framework [34], we propose three promising strategies to address the barriers to adoption identified by this study (Figure 2).

#### Promoting Trust in mHealth Solutions

Findings of this study showed that both participants with SCI and health care professionals had concerns about the data protection and privacy of the app. Individuals with SCI in particular were worried that personal health information might be shared with third parties without their explicit consent. These findings indicate that trust in the app’s commitment and adherence to data protection and privacy plays an important role when it comes to adoption. In this context, transparency plays a central role [35,36].

To create trust in an app’s commitment and adherence to data protection, users need to be informed about the kind of data being collected, ownership of and control over the data, and their right to have their data deleted. All this information needs to be accessible to prospective users in a clear and concise format, informing them about their rights to access and control their data. However, how personal information is handled by mobile health apps often remains unclear [37-39]. Some apps, for example, track and share user data continuously by default, even when the app is not in active use, sometimes without explicitly informing users about these tracking practices in their terms of use [39]. A recent study [36] that investigated end users’ opinions on what constitutes a trustworthy mobile health app found that users interpreted lengthy terms of service and privacy policies as an attempt by developers to obscure possible risks. Moreover, the study showed that users perceived apps that request “too much” personal information as untrustworthy.

Tools like the mHealth App Trustworthiness checklist [36] can help to guide developers in creating trustworthy apps to foster the adoption of mHealth solutions. Visualization techniques might be promising approaches to make privacy information more accessible, making it easier for users to understand how apps collect, use, and share data. Also, a privacy by design approach that allows users to opt in for data sharing and enables granular permissions could help to strengthen user privacy [39] and, as a result, foster trust. Last but not least, the credibility of the provider (ie, the organization behind the app) will also likely have an impact on prospective users’ trust. To further strengthen prospective users’ trust, authors have also proposed to include logos of reputable organizations endorsing the app (eg, universities or hospitals) [40].

#### Providing a Personalized User Experience

Our findings showed that established attitudes and behavioral patterns of individuals with SCI constitute potential barriers to adoption. “Why use an app to contact a health care professional when I can just WhatsApp them?” “Why would I need a reminder to check my skin after having done it for the past 20 years?” “Why do I need the app? I never had problems with my skin.” These are the kind of questions prospective users might ask themselves. All of these questions hint at an important aspect: value. How can we create value for a diverse user group ranging from newly injured individuals to experienced wheelchair users, from those that constantly struggle with pressure injuries to those who have never experienced any problems with their skin? This is where personalization comes in.

Personalization technologies can help to better tailor the self-management app to an individual user’s needs and preferences [41] by ensuring that recommendations regarding preventive measures are perceived as relevant and actionable rather than generic advice. In matching an individual’s preferences and lifestyle, personalization can lower barriers to act on recommendations. Contextualization can ensure that interventions (eg, in the form of recommendations) are delivered at moments of need (eg, long time with low activity levels) or at an opportune moment when a particular recommendation is easy to follow (eg, when leaving work) [42,43]. While personalization is a familiar concept in health communication, artificial intelligence and machine learning techniques offer unprecedented opportunities to take personalization to the next level [42,44,45]. These techniques can then be used to translate population-level data alongside individual user data to provide a personalized user experience, similar to recommendation systems like Netflix [41,42]. However, for personalization to be realized, users are required to disclose personal information [46]. Such information may include personal information that users actively enter themselves (eg, age, gender) but it may be complemented by information on app usage through tracking (eg, how often or when the app is used).

It is, however, important to acknowledge the limits of personalization. What personalization likely cannot achieve is motivating download and first-time use. This is where health care professionals have to step in to promote and endorse the app. However, in order to encourage health care professionals to endorse the app, it is first important to obtain their buy-in. To this end, it will be important to demonstrate that the app allows health care professionals to better support their patients in effectively reducing pressure injuries (ie, by supporting self-management and early detection). Moreover, it will be essential to show that the app will not impose an additional burden on health care professionals, leading to increased workloads.

#### Ensuring Organizational Support and Interoperability

It is important to consider that in the case of an app that enables communication between patients and health care providers, there are two prospective user groups: senders and receivers. Even though many of the app’s features can be used by people with SCI independently, the communication component requires uptake from health care professionals as well. Findings of our study indicate that health care professionals were particularly concerned about challenges in integrating the proposed solution with currently used systems and worried about additional workload to reenter existing information. Additionally, there were also concerns about inappropriate use of the app that could lead to overburdening of staff.

Given the crucial role of health care professionals’ acceptance of new technologies, it is of utmost priority to take these concerns seriously and to respond to them in an adequate manner. In addition to ensuring the technical requirements are met, including issues related to system operability, organizations also need to ensure that new systems can be smoothly integrated into existing workflows [47]. Organizations may also want to establish technical training and support to deal with cases of malfunction or inappropriate use [48]. This would allow health care professionals to feel that support is available and may help to reduce concerns over increased workloads.

Close to the completion of our own project, we realized that we had invested a lot of time and resources to understand what the user interface (ie, the front end of the application that individuals with SCI would interact with) should look like. However, our findings indicated that we had neglected to consider the administrative interface (ie, the back end of the application operated by health care professionals). In other words, we had not collected sufficient information on the internal workflows of the care provider Parahelp to determine whether and how the app could actually be integrated into their daily practice. In the current app prototype, we also did not consider reimbursement aspects, which constitute a relevant factor for adoption but, at the same time, trigger many other organizational questions.

### Limitations

This study is subject to some limitations. First, we need to acknowledge that our research was focused on a narrow, specialized health care setting in Switzerland. However, while our findings may not necessarily be directly applicable to other health care settings or health conditions, they have relevant implications for mHealth research more broadly. In particular, the strategies we identified to promote the adoption of evidence-based mHealth solutions lend themselves to inform research on self-management apps in other areas. Second, two focus group participants (both affiliated with Parahelp) had been involved in the co-design process of the app prototype at an earlier stage of the project. This may have led them to hold more favorable attitudes toward the project and possibly steer the focus group discussion in this direction. To account for this potential bias, we made it clear that critical scrutiny would help us to identify potential weaknesses of the app prototype, which would in turn foster improvement. Lastly, we need to acknowledge that the characteristics of the wheelchair users with SCI who participated in this study may have had an impact on our findings. While we aimed for a balanced sample of wheelchair users in terms of age, gender, and lesion level, we cannot rule out the possibility that participating individuals may have had overall more favorable attitudes toward mHealth solutions than those who did not take part. While findings of this study may not reflect the views of the Swiss population with SCI, they provide the basis for further investigations at a population level.

### Conclusion

Findings of this study indicate that even though co-design seems to be a promising approach to develop self-management tools that meet the needs of different stakeholder groups, it does not guarantee adoption. To fully harness the potential of a co-designed mHealth self-management solution and to overcome barriers to adoption, further efforts are needed. In particular, there is a need to foster trust in mHealth solutions among prospective users, including both patients and health care professionals. Moreover, increasing the personal relevance of mHealth solutions through personalization may be a promising approach to driving adoption. However, with data-driven approaches, such as machine learning, several ethical questions need to be considered, including but not limited to issues of autonomy, data protection, and privacy. Last but not least, organizational support and financial concepts also play a key role in mHealth adoption, particularly in the clinical context.

## Appendix

Multimedia Appendix 1 Screenshots of the self-management app prototype.

## Abbreviations

FG1: focus group 1
FG2: focus group 2
mHealth: mobile health
SCI: spinal cord injury
