# Supplementary material for: Opportunities and Challenges of a Self-Management App to Support People With Spinal Cord Injury in the Prevention of Pressure Injuries: Qualitative Study
Source: JMIR Mhealth Uhealth. 2020 Dec 9;8(12):e22452. doi: 10.2196/22452 (PMC7758166; doi:10.2196/22452)
Supplement: Multimedia Appendix 1 [file mhealth_v8i12e22452_app1.pdf]

Multimedia Appendix 1

Screenshots of the self-management app prototype

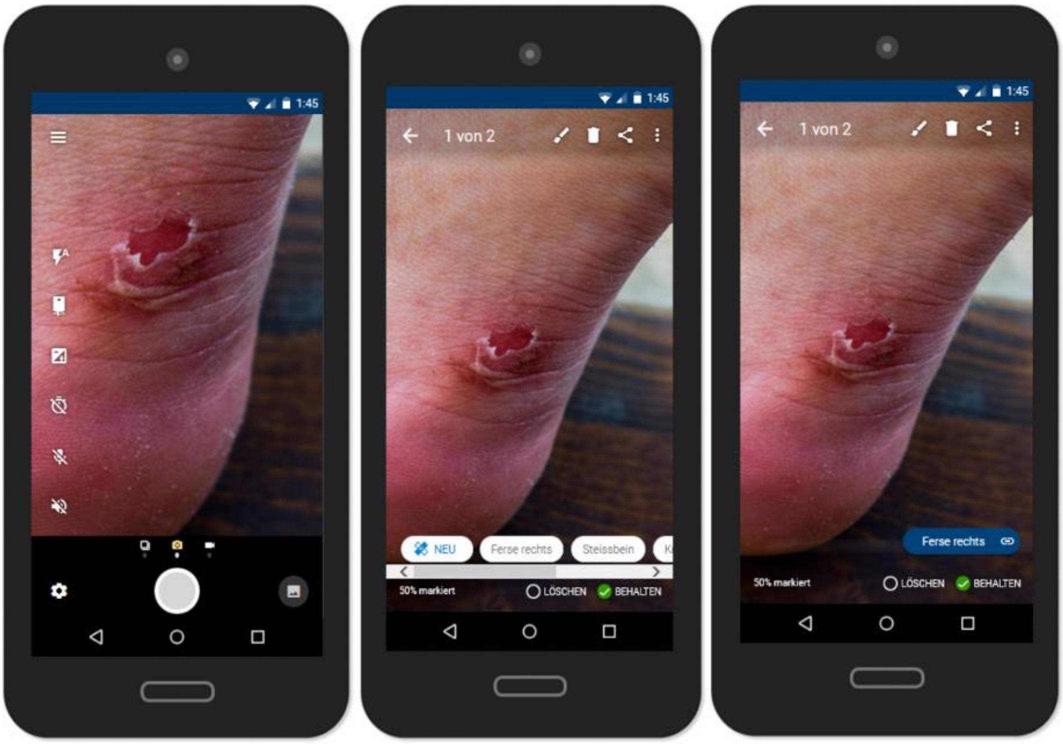

Figure 2 Smart camera

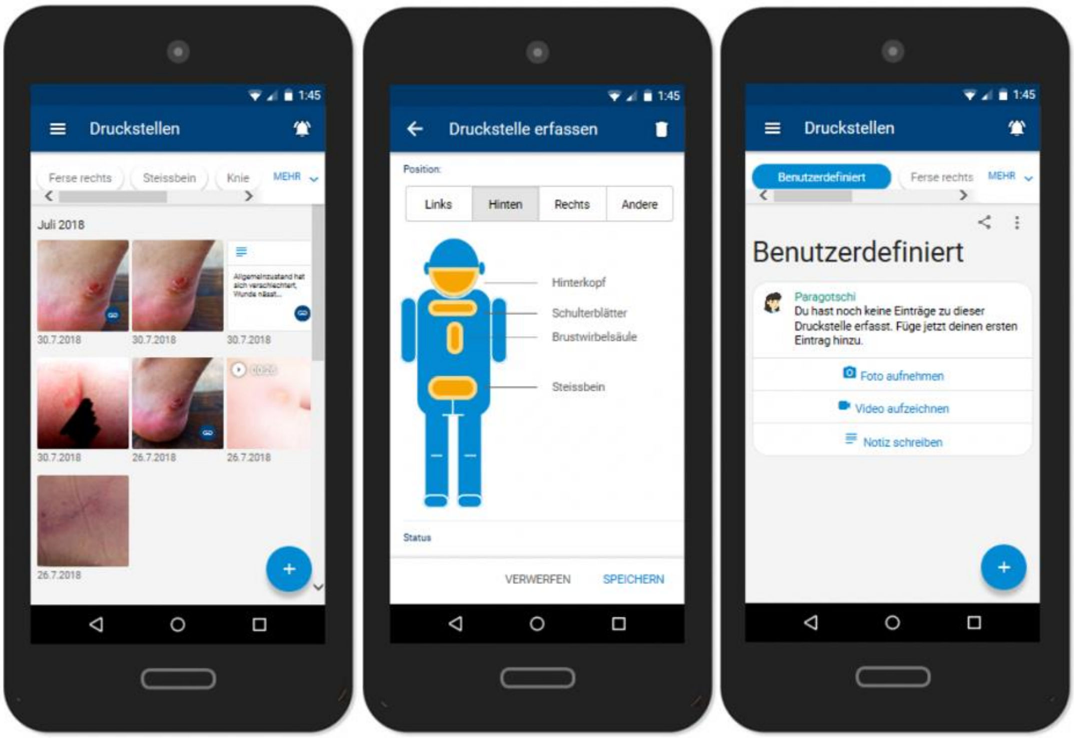

Figure 1 Pressure injury diary

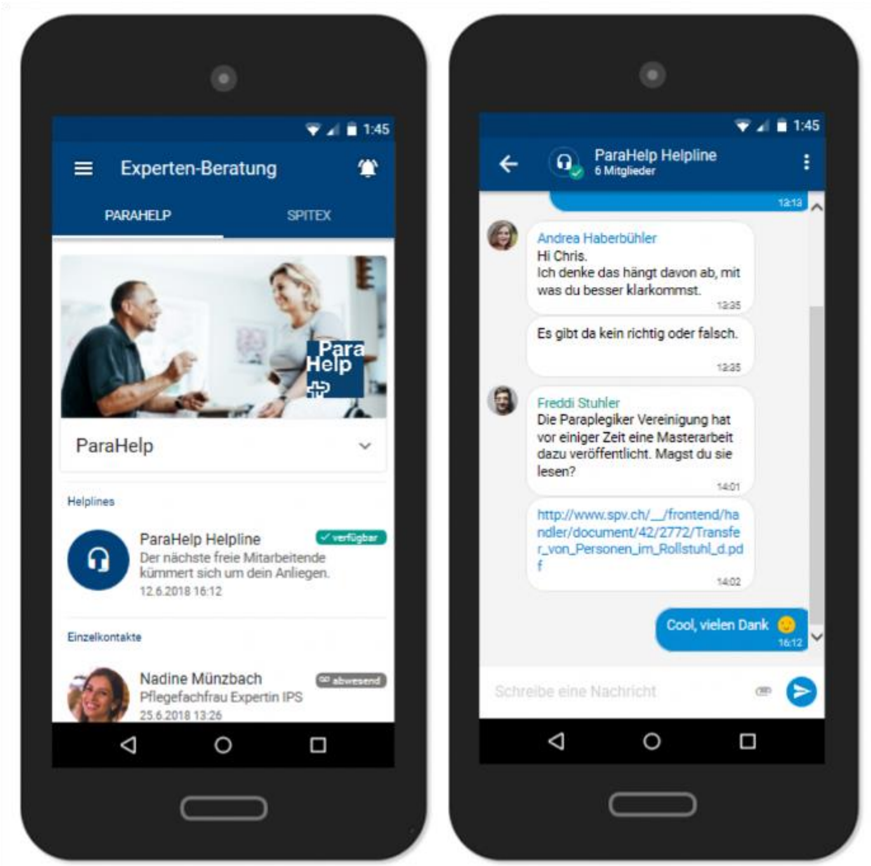

Figure 3 Expert consultation

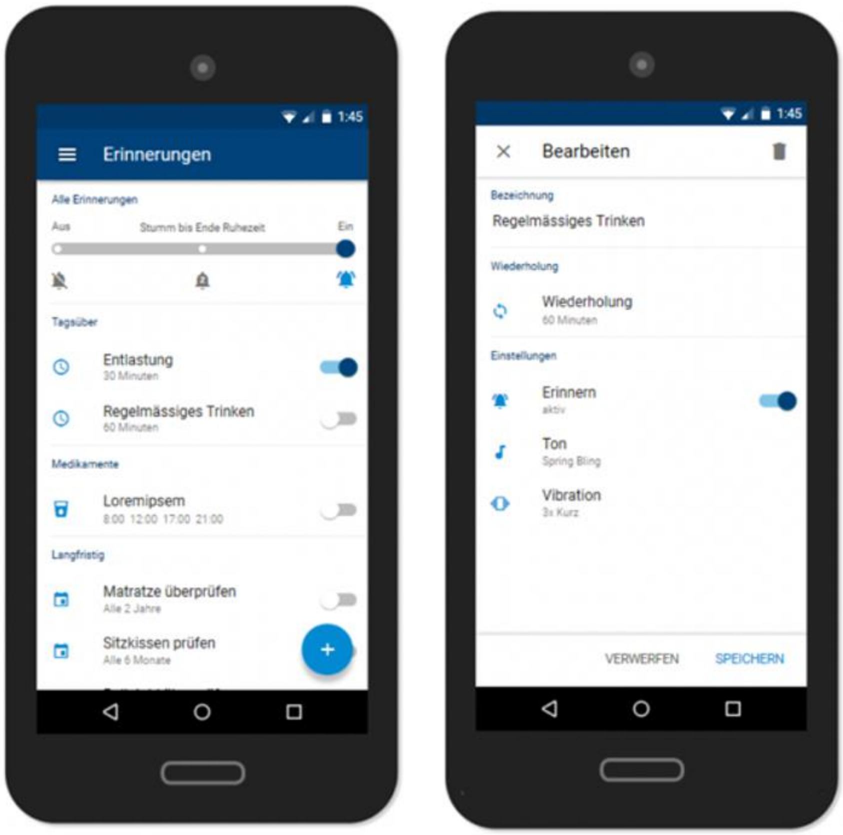

Figure 4 Reminders

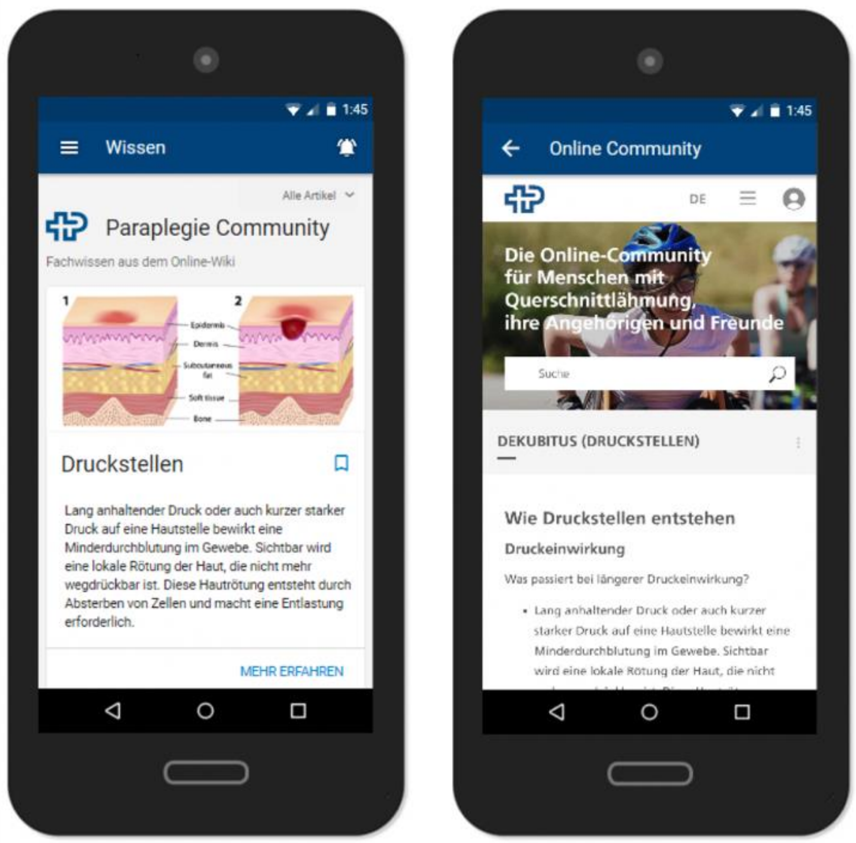

Figure 5 Knowledge repository
